# Association of indoor dust microbiota with cognitive function and behavior in preschool-aged children

**DOI:** 10.1186/s40168-022-01406-9

**Published:** 2023-01-02

**Authors:** Yinthe Dockx, Martin Täubel, Janneke Hogervorst, Leen Luyten, Martien Peusens, Leen Rasking, Hanne Sleurs, Katrien Witters, Michelle Plusquin, Maria Valkonen, Tim S. Nawrot, Lidia Casas

**Affiliations:** 1 Centre for Environmental Sciences, Hasselt University, Agoralaan Building D, 3590 Diepenbeek, Belgium; 2 Environmental Health Unit, Department Health Security, Finnish Institute for Health and Welfare, Kuopio, Finland; 3 Center for Environment and Health, Department of Public Health, Leuven University (KU Leuven), Herestraat 49–706, BE-3000 Leuven, Belgium; 4 Social Epidemiology and Health Policy, Department of Family Medicine and Population Health, University of Antwerp, Doornstraat 331, 2610 Wilrijk, Belgium; 5 Institute for Environment and Sustainable Development (IMDO), University of Antwerp, Groenenborgerlaan 171, 2020 Antwerp, Belgium

**Keywords:** Microbiota, Indoor, Diversity, Childhood, Cognition, Behavior

## Abstract

**Background:**

Childhood cognitive development depends on neuroimmune interactions. Immunomodulation by early-life microbial exposure may influence neuropsychological function. In this study, we investigate the association between residential indoor microbiota and cognition and behavior among preschoolers.

**Results:**

Indoor-settled dust bacterial and fungal characteristics were assessed using 16S and ITS amplicon sequencing (microbial diversity) and qPCR measurements (microbial loads). Child behavior was assessed using four scales: peer relationship, emotional, conduct, and hyperactivity was assessed by the Strengths and Difficulties Questionnaire (SDQ). Cognitive function was assessed using four tasks of the Cambridge Neuropsychological Test Automated Battery (CANTAB) software. The first two tasks were designed to assess attention and psychomotor speed (Motor Screening (MOT) and Big/Little Circle (BLC)) and the last two to evaluate the child’s visual recognition/working memory (Spatial Span (SSP) and Delayed Matching to Sample (DMS)). Among the 172 included children (age 4–6 years), we observed a 51% (95%CI;75%;9%) lower odds of children scoring not normal for hyperactivity and a decrease of 3.20% (95%CI, −6.01%; −0.30%) in BLC response time, for every IQR increase in fungal Shannon diversity. Contrarily, microbial loads were directly associated with SDQ scales and response time. For example, a 2-fold increase in Gram-positive bacterial load was associated with 70% (95%CI 18%; 156%) higher odds of scoring not normal for hyperactivity and an increase of 5.17% (95%CI 0.87%; 9.65%) in DMS response time.

**Conclusions:**

Our findings show that early-life exposure to diverse indoor fungal communities is associated with better behavioral and cognitive outcomes, whereas higher indoor microbial load was associated with worse outcomes.

Video Abstract

**Supplementary Information:**

The online version contains supplementary material available at 10.1186/s40168-022-01406-9.

## Background

During childhood, environmental factors can readily induce adaptive changes with permanent health effects in later life [1, 2]. Cognition-related outcomes are particularly interesting during childhood, because neural connections are developing and maturing, making them highly receptive to external factors [3, 4]. Considering that children spend most of their time indoors and particularly within their own home environment [5], it is important to investigate the early-life exposure to components present in the child’s home, including the indoor microbiota represented in house dust. There is a good number of studies providing consistent evidence for a connection between indoor dust microbiota and other health outcomes, in particular regarding the development of asthma [6–10]. Immunomodulation by microbiota in the course of early childhood exposure as part of the immune development is proposed as a potential pathway explaining these associations [11–14]. Interestingly, in addition to being a critical period for the maturation of the immune system, childhood is also crucial to neural development and neuroimmune interactions are thought to play a regulating role in synaptic plasticity [15]. This close connection is, for example, apparent by the fact that attention-deficit/hyperactivity disorder (ADHD) symptoms oftentimes coincide with allergies or asthma symptoms [16–18], and increased inflammation has been linked to poorer cognitive performance [19–21]. Consequently, it is important to explore further the association between indoor microbiota and childhood neuropsychological function.

To date, only few studies [22–25] have investigated the potential role of early-life exposure to indoor microbial communities and their indoor determinants in cognitive function and behavior. Overall, the results of these studies consistently showed that the presence of mold and dampness at home was associated with poorer cognitive function and behavioral problems during childhood [22, 23, 25]. Another study showed that occasional farm animal contact was associated with better cognitive function at the age of 4 years. The authors considered exposure to microbial agents as one potential explanation for this finding [23]. So far, only one study has included actual measures of indoor microbial diversity. In this study [24], it was found that early-life indoor fungal diversity exposure was associated with higher odds of hyperactivity/inattention for 10-year old children but lower odds at the age of 15 years. In the present study, we complement the results of existing studies by investigating the associations of behavioral and cognitive function with indoor microbial exposure, assessed qualitatively and quantitatively, in young children aged 4–6 years, participating in the ENVIR*ON*AGE birth cohort.

## Methods

### Study design

ENVIRonmental influence *ON* early AGEing (ENVIR*ON*AGE) is a Belgian birth cohort that started in 2010, with ongoing recruitment for mother-newborn pairs at birth in the East-Limburg Hospital (Genk, Belgium). Complete information on the eligibility and recruitment process is presented elsewhere [26]. A follow-up examination is conducted when the child is 4–6 years, where parents fill out questionnaires to provide lifestyle and socio-demographic information, and the Strengths and Difficulties Questionnaire (SDQ) to assess the child’s behavior. In addition, during this visit, the child performs cognitive testing using the Cambridge Neuropsychological Test Automated Battery (CANTAB) [27]. The study protocol was approved by the ethical committee of Hasselt University and complied with the Helsinki Declaration. Parents gave written informed consent and children verbal permission [26, 28]. This study followed the Strengthening Reporting of Observational Studies in Epidemiology (STROBE) reporting guideline.

In 2017 and 2018, a subset of the ENVIR*ON*AGE participants were asked to participate in home visits. More specifically, we selected households that already participated in the follow-up examination or had the examination planned close to the home visits, did not move in between, and had no indoor renovations planned. In total, 233 of the 284 eligible households were contacted, of which 189 accepted to participate, resulting in a participation rate of 81% (Supplemental Fig. 1). Due to logistical constraints, eight samples were not collected. Additionally, we excluded two samples because of sampling irregularities, two samples with less than 1000 sequences due to insufficient dust and one sample because sampling period exceeded the predetermined maximum of 9 weeks. Of the 176 children with appropriate dust samples, 171 and 172 children completed the SDQ and CANTAB, respectively.

### Indoor microbial assessment during home visit

Settled dust was collected using two sterile, open-faced Petri dishes (92x16mm) over a period of minimum four and maximum nine weeks (mean 43.4 days), in spring 2017 and spring 2018, in the household’s living room. They were placed approximately 2 m above floor level, a safe distance from major air flows and heating sources [29]. Upon collection, the Petri dishes were sealed and stored at −20°C to be processed in the summer of 2018, as described previously [30]. After processing, samples were shipped frozen on dry ice to the Finnish Institute for Health and Welfare (Kuopio, Finland), where DNA extraction was conducted as described earlier [30], storing the DNA at −20°C until sequencing.

Extracted DNA from dust and control samples was shipped frozen to the sequencing service partner LGC Genomics (Germany) for library preparation and sequencing. For bacteria, the V4 region of the bacterial 16S rRNA gene was amplified using 515F/806R primers [31]. For fungi, the Internal Transcribed Spacer region 1 (ITS1) was amplified using ITS1F/ITS2 primers [32].

The PCR procedure, sequencing, sequence processing, and bioinformatics analyses are described more in-depth in supplement. In brief, 16S and ITS amplicon data was processed by standard dada2 pipeline version 1.8 [30]. Taxonomy was assigned using SILVA [33] database version 132 for bacteria and UNITE database version 7.2 for fungi [34]. Downstream processing included removal of chimeras, chloroplast, and mitochondria sequences, as well as of potential contaminants utilizing negative controls and Decontam package version 1.2 [35].

QIIME software version 1.9.1 [36] was used to calculate Chao1 richness and Shannon diversity index using rarefaction values of 1495 and 3956 sequences for bacteria and fungi, respectively. The Chao1 index is an abundance-based estimator of species richness. The Shannon diversity index incorporates species evenness, i.e., the homogeneity of species abundance, with species richness.

We used quantitative PCR to calculate the total Gram-negative and Gram-positive bacterial and fungal loads, as described previously [30]. We determined the number of microbial cell equivalents (CE) in the samples using relative quantification, utilizing an internal standard to adjust for the presence of DNA inhibitors and/or variability in DNA extraction efficiency [37]. Results were normalized for sampling surface area and accumulation duration and expressed as CE per m^2^ settling surface area per day, referred to hereafter as microbial load.

### Neuropsychological assessment during follow-up visit

#### Behavioral outcomes

To assess the child’s behavior, parents filled out the SDQ [38], a validated screening method for psychiatric disorders in children [39–41]. From this questionnaire, four scales (range 0–10) are calculated from five statements each: peer relationship, emotional, conduct, and hyperactivity and were additionally combined to calculate a Total Difficulties Score (range 0–40). We used British cutoff guidelines and categorized SDQ outcomes into discrete variables by grouping borderline and abnormal scores together to define a “not normal” category. Being not normal was defined when scores were equal or above 3 for peer relationship, 4 for emotional, 3 for conduct, 6 for hyperactivity, and 14 for the Total Difficulties Score [42].

#### Cognitive function outcomes

**Fig. 1:**
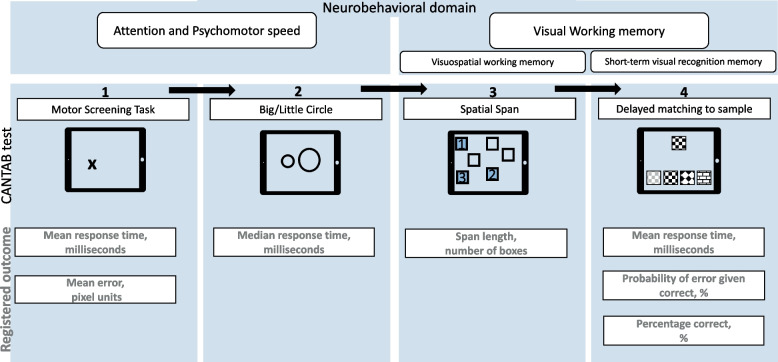
Schematic representation of the cognitive measurements. The four tasks of the Cambridge Neuropsychological Test Automated Battery (CANTAB) are shown with their corresponding domain and registered outcome variables. Arrows indicate the sequence of the test. While the child was administered the cognitive CANTAB tasks, the SDQ was filled out by the accompanying parent

Cognitive function was assessed via CANTAB [27] software on a touchscreen tablet, reliable for measuring executive functions in young children [43]. In total, each child had to complete four tasks (Fig. 1). Two tasks assessed the attention and psychomotor speed (Motor Screening Task (MOT) and Big/Little Circle (BLC)), and two assessed the visual working memory (Spatial Span (SSP) and Delayed Matching to Sample (DMS)). Detailed information on cognitive assessment is provided in supplement.

The MOT outcome variables included the response time and error. The response time is the average time in milliseconds to select the cross successfully. The error is the average distance in pixel units between the child’s press and the cross’s center on all successful trials. Low values in response time and in erro*r* indicate better performance. The BLC outcome variable was response time, being, in this test, the median time in milliseconds to select the right circle successfully, low values indicate better performance. The SSP measured the maximum sequence length the child could correctly recall, further referred to as span length, higher considered better. In the fourth and last test, i.e., DMS, the first outcome variable was the average time, in milliseconds, it took to correctly answer on the first try, further referred to as response time; lower values considered better. We excluded response times calculated on less than 25% of the trials. The last outcome variables were the probability of error if the previous trial was correct, and the total proportion of correct answers on first try, further referred to as percentage correct, both expressed in percentages. A lower probability of error and higher percentage correct are considered better.

### Covariables

During the follow-up examination, questionnaires were used to collected lifestyle and clinical information. Maternal education was used to represent socioeconomic status, coded as “low” (no diploma or primary school), “middle” (high school), or “high” (college or university degree). In addition, we obtained information on average daily screen time, defined as watching television, playing computer games and tablet use, categorized as “<1 h per day,” “1–2 h per day,” and “>2 h per day.” The time of examination was used as a continuous variable in the main analyses and categorized in the sensitivity analysis into morning (before 12pm), early afternoon (from 12pm up to 4pm), and late afternoon (after 4pm). Additionally, we obtained information on parental smoking coded dichotomously as non-smoking parents versus one or both parents smoking.

The residential addresses of the households were geocoded and categorized into rural, and suburban or urban, based on population density, employment, location, and spatial planning of statistical sectors (Flemish Government-Department Environment). In addition, we calculated average black carbon (BC) exposure by averaging daily BC concentrations at the residential address over the sampling period, using a spatiotemporal interpolation method [44] as described elsewhere [30]. Additionally, average outdoor temperature (°C), provided by the Belgian Royal Meteorological Institute, was calculated as the daily mean temperatures measured at a representative measuring station (Diepenbeek, Belgium) averaged over the sampling period.

Upon sample collection, we obtained additional household information: number of household members, pet ownership, ventilation type, and sampling duration. Pet ownership was dichotomized into the presence of a furry pet (cat, dog, rabbit, hamster, or guinea pig) or not and ventilation type into the use of passive ventilation or not.

### Statistical analysis

For the statistical processing, we used the R environment version 3.6.0 [45]. We screened for outliers using a threshold of more or less than three times the standard deviation away from the mean. We removed two outliers for the bacterial Shannon diversity index. For the Gram-negative, Gram-positive, and fungal load, we removed two, five, and three outliers, respectively, and log-transformed values (base 10) to better comply with linear model assumptions. We identified certain core variables to be included in all of our models, including child’s age, sex, maternal education, urbanicity, and sampling duration, which reflect important clinical, socioeconomical, and technical information regarding the microbial and cognitive assessment.

To examine the associations between microbial exposure and the child’s behavior, we used logistic regression models for the SDQ outcomes, adjusting for the aforementioned core variables (child’s age, sex, maternal education, urbanicity, and sampling duration), as well as the number of household members, which was identified as an additional potential confounder for this analysis. Results regarding microbial diversity are expressed as odds ratios (OR) per interquartile range (IQR) increase in microbial diversity indices or for a 2-fold increase in microbial load.

To investigate microbial exposure in association with cognitive CANTAB outcomes, we log-transformed (base 10) all response times to better comply with assumptions on model linearity. Furthermore, we performed multivariable linear regression models adjusting for the aforementioned core variables (child’s age, sex, maternal education, urbanicity, and sampling duration), as well as the time of examination, reflecting relevant technical information regarding the CANTAB outcomes. Results are expressed as a unit change or a percentage change for the log-transformed response time outcomes (with 95% CI), per IQR increment in microbial diversity or per 2-fold increase in microbial load.

To assess the robustness of our findings, we additionally adjusted for potential confounders: smoking, determinants for indoor microbiota (average outdoor temperature, BC exposure, pet ownership, and ventilation), screen time (as a proxy for prior screen familiarization), and number of household members. We performed two sensitivity analyses for CANTAB models, excluding children who showed any signs of possible disinterest during cognitive testing, based on behavioral remarks and irregular touch patterns (*n*=1, 4, 7, and 5 for MOT, BLC, SSPM and DMS, respectively). Lastly, because a child’s performance may depend on tiredness, we restricted our analysis to children that performed the cognitive tests before 4pm.

## Results

### Study population

Descriptive statistics on follow-up examination, household, and child characteristics are presented in Table 1. The majority of follow-up examinations were performed in the afternoon (56.2%), and half of the children were boys and were on average 4.5 years old (± 0.3) and two thirds of the mothers reporting having high education.

**Table 1:** Characteristics of the households and mother-child pairs that performed the SDQ and CANTAB tests

**Characteristics of the follow-up examination**	
Time of examination	
Morning (before 12 pm)	68 (39.3)
Afternoon (from 12 to 4 pm)	97 (56.1)
Late afternoon (after 4 pm)	8 (4.6)
Season of examination	
Spring	47 (27.2)
Summer	44 (25.4)
Autumn	43 (24.9)
Winter	39 (22.5)
**Characteristics of the child**	
Age at follow-up, years	4.5 ± 0.3
Sex, boy	87 (50.3)
Time spent watching television/playing games	
None to less than 1 hour/day	57 (32.9)
1-2 hours/day	96 (55.5)
>2 hours/day	14 (8.1)
Missing information	6
**Characteristics of the mother**	
Age of the mother at follow-up visit, years	35.1 ± 3.8
Educational level	
Low (no high school diploma)	5 (2.9)
Middle (high school diploma)	38 (22.0)
High (college degree or higher)	130 (75.1)
**Characteristics of the household**	
Smoking	
Non-smoker parents	128 (74.0)
One or both parents smoked	36 (20.8)
Missing information	9
Number of household members	4 ± 0.8
Presence of furry pets, yes	82 (47.1)
Passive ventilation, yes	140 (80.5)
**Characteristics of the dust sampling**	
Age of child, during sampling	4.8 ± 0.6
Duration of sampling (days)	43.4 ± 5.6
Sampling average outdoor temperature (°C)	16.7 ± 1.9
Sampling average ambient airborne BC concentration (μg/m^3^)	0.7 ± 0.2

Continuous variables presented as mean ± sd and categorical variables as *n* (%)

The SDQ and CANTAB outcomes are summarized in Table 2. The largest group of children scoring not normal occurred within the emotional and conduct scales, where approximately a quarter of the children scored not normal, followed by the hyperactivity scale (17.5%). In addition, the response times of the CANTAB tests assessing the attention and psychomotor speed (i.e., MOT and BLC) correlated the strongest (Supplemental Fig. 2).

**Table 2:** Description of the behavioral (SDQ) and cognitive (CANTAB) outcomes and microbial measurements

	n (%)	min	P25	P50	P75	max
**SDQ outcomes**						
Peer relationship scale						
Normal	143 (83.6)					
Not normal	28 (16.4)					
Emotional scale						
Normal	126 (73.7)					
Not normal	45 (26.3)					
Conduct scale						
Normal	127 (74.3)					
Not normal	44 (25.7)					
Hyperactivity scale						
Normal	141 (82.5)					
Not normal	30 (17.5)					
Total difficulties score						
Normal	143 (83.6)					
Not normal	28 (16.4)					
**Cantab outcomes**						
**Attention and psychomotor speed**						
MOT						
Response time, ms	170	659.3	817.8	980.4	1161.1	2961.8
Error, pixel units	170	8.2	12.0	13.89	15.99	21.5
BLC						
Response time, ms	172	726	898.6	985.0	1083.6	1467.0
**Visual and working memory**						
SSP						
Span Length, number of boxes	172	0	2	3	3	5
DMS						
Response time on first try, ms	143	1340.0	3303.9	4169.9	5368.7	13994.7
Error given correct answer, %	169	13.3	41.7	54.6	66.7	100.0
Percentage correct, %	169	10.0	35.0	45.0	55.0	85.0
**Microbial measurements**						
**Microbial diversity**						
Bacteria						
Chao1 richness	173	111	294	406	501	728
Shannon diversity	171	4.93	6.57	7.11	7.61	8.57
Fungi						
Chao1 richness	172	24	92	131	174	300
Shannon diversity	172	0.66	2.71	3.40	4.09	5.57
**Microbial load**						
Gram-negative bacterial load	171	9683	150635	289297	486076	2786787
Gram-positive bacterial load	168	437	84846	157486	273577	1010599
Fungal load	170	155	20332	33724	62577	211855

An overview of the microbial variables is also provided in Table 2. Overall, indoor dust bacterial diversity and load were found to be higher than fungal measures. The microbial diversity indices were overall positively and strongly correlated with each other. In contrast, microbial load was overall negatively correlated with the corresponding microbial diversity indices (Supplemental Fig. 3).

### Associations between behavior and indoor microbiota

After adjustment, we generally observed tendencies for inverse associations between the SDQ outcomes and microbial diversity indices (Table 3). These associations were stronger for hyperactivity and the Total Difficulties Score and only statistically significant for the fungal Shannon diversity index. More specifically, an IQR increase in the fungal Shannon diversity was associated with a 51% (95%CI 75%; 9%) and a 57% (95%CI 80%; 15%) lower odds of a not normal score for hyperactivity and Total Difficulties Score, respectively. In contrast, microbial loads were directly and significantly associated with hyperactivity and the Total Difficulties Score. A 2-fold increase in bacterial (Gram-negative and Gram-positive) and fungal loads was associated with a significant increase in the odds of scoring not normal for hyperactivity. In addition, a 2-fold increase in Gram-negative and Gram-positive bacterial load was associated with a 55% (95%CI 7%; 130%) and a 46% (95%CI 1%; 120%) increased odds of children scoring not normal for total difficulties.

**Table 3:** Adjusted^a^ associations (OR and 95%CI) of microbial diversity indices and loads with behavioral problems, *n*=171

	Diversity indices^b^				Microbial load^c^		
	Bacteria		Fungi		Gram-negative load	Gram-positive load	Fungal load
	Chao1	Shannon	Chao1	Shannon			
Peer relationship scale	0.86 [0.44;1.64]	0.91 [0.52;1.63]	1.16 [0.60;2.21]	1.03 [0.53;2.00]	1.32 [0.93;1.92]	1.39 [0.97;2.05]	1.11 [0.80;1.59]
Emotional scale	0.81 [0.47;1.39]	1.06 [0.65;1.74]	1.13 [0.65;1.94]	0.82 [0.47;1.41]	0.95 [0.71;1.27]	0.97 [0.75;1.29]	0.88 [0.67;1.15]
Conduct scale	0.80 [0.47;1.35]	0.71 [0.44;1.15]	0.68 [0.40;1.15]	0.68 [0.40;1.14]	1.10 [0.84;1.45]	0.96 [0.74;1.24]	1.19 [0.92;1.59]
Hyperactivity scale	0.66 [0.35;1.22]	0.82 [0.47;1.44]	0.61 [0.31;1.14]	**0.49 [0.25;0.91]**	**1.54 [1.09;2.22]**	**1.70 [1.18;2.56]**	**1.67 [1.15;2.53]**
Total difficulties Score	0.61 [0.31;1.16]	0.70 [0.40;1.24]	0.72 [0.35;1.39]	**0.43 [0.20;0.85]**	**1.55 [1.07;2.30]**	**1.46 [1.01;2.20]**	1.34 [0.94;1.98]

^a^Associations adjusted for child’s sex and age, maternal education, sampling duration, number of household members, and urbanicity. Estimates presented in bold are statistically significant *p*<0.05

^b^Associations with microbial diversity indices are expressed for IQR increments

^c^Associations with microbial load are expressed for a 2-fold increment

### Associations between cognitive outcomes and indoor microbiota

**Fig. 2:**
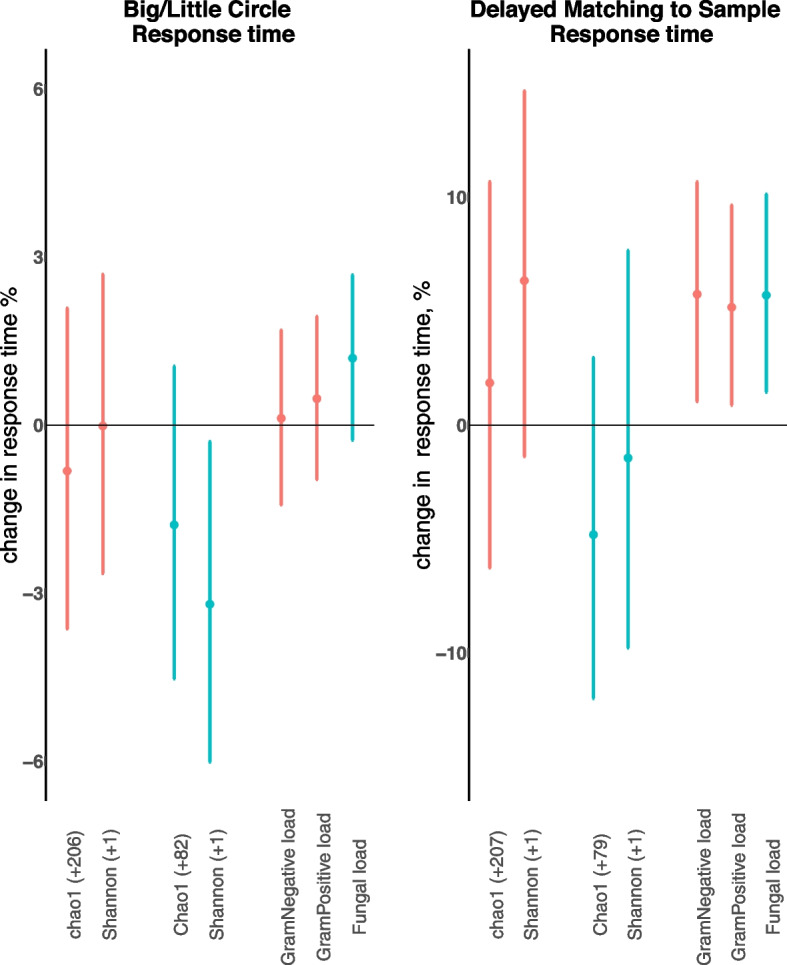
Overview of the statistically significant associations between microbial diversity and load and cognitive CANTAB outcomes. The association is shown between bacterial  and fungal  diversity indices (Chao1 richness and Shannon index) and Gram-negative bacterial, Gram-positive bacterial, and Fungal load and two cognitive CANTAB latency outcomes (Big/Little Circle (BLC) assessing attention and psychomotor speed and Delayed Matching to Sample (DMS) assessing visual recognition memory). Regression coefficients and 95% CI are given in units or as percentage change and expressed for an IQR increase in microbial diversity, or for 2-fold increase in microbial load. Diversity-specific IQRs are given in the *x*-axis. Models were adjusted for child’s sex, age, maternal education, time of examination, sampling duration, and urbanicity

After adjustment, we found for an IQR increase in indoor fungal Shannon diversity a decrease of 3.2% (95%CI, −6.0%; −0.3%) in the BLC response time, assessing attention and psychomotor speed. In contrast, we found for a 2-fold increase in Gram-negative bacterial, Gram-positive bacterial, and fungal loads, an increase of 5.74% (95%CI 1.04; 10.67), 5.17% (95%CI 0.87; 9.65), and 5.70% (95%CI 1.44; 10.14), respectively, in the DMS response time, assessing visual recognition memory. Other association estimates were not statistically significant. The statistically significant results are presented in Fig. 2, and the exact values of the association estimates are provided in Supplemental Table 1.

### Sensitivity analyses

Additionally, adjusting for smoking, screen time, number of household members, or determinants for indoor microbiota did not importantly change the observed associations (data not shown). In addition, excluding children who showed signs of possible disinterest during CANTAB testing (Supplemental Table 2), and children that performed these tests after 4pm did not result in important changes to the observed associations.

## Discussion

In this study, we used qualitative and quantitative measures to characterize early-life indoor dust microbial exposure and evaluated associations with child behavior and cognitive function in four to 6-year-old children. One key finding was that higher indoor fungal diversity was associated with a reduction in hyperactive behavioral traits and the Total Difficulties Score. In contrast, exposure to higher quantities, i.e., loads of microbiota indoors were generally associated with worse outcomes in these behavioral assessments. For cognitive function, we obtained similar results. Here, higher fungal diversity was associated with shorter (better) BLC response time, whereas higher indoor microbial loads were associated with longer (worse) DMS response times.

To date, only few studies have investigated associations between behavioral and cognitive outcomes and indoor microbiota measures, with the reported results being inconsistent [22–25]. For example, in a German cohort [24], heterogenous associations were described between the indoor microbial diversity and the development of hyperactive problems during childhood. The authors reported that early-life indoor fungal diversity exposure was associated with higher odds of hyperactivity/inattention for 10-year old children but lower odds at the age of 15 years. Interestingly, similar differences in associations with fungal exposures depending on age have been observed for atopic outcomes, where fungal diversity seems to have a protective effect in early childhood, whereas the effects are attenuated in later life [46]. In addition to improved behavioral outcomes, we found indoor fungal diversity to be associated with improved response time for BLC, related to the neurological domain of attention and psychomotor speed. As such, a reduction in response time corresponds with improved attention, making this association compatible with the observed reduction in hyperactivity/inattention problems of the SDQ [47].

In contrast, indoor microbial quantity was found to be associated with an increased likelihood of a child having hyperactivity problems and Total Difficulties, in addition to an observed increase in the response time for DMS. Though different from the neurological domain of attention and psychomotor speed, the visual recognition memory, which in turn corresponds strongly with visual working memory [48–50], is innately attention-driven and thus an increase in response time is compatible with decreased attention [51]. Our results suggests that, similar to immune-related health outcomes, early-life exposure to a higher fungal diversity is associated with improved cognitive outcomes, whereas exposure to higher microbial quantities is associated with decreased cognitive function [52–54]. In our study, we mainly see an association between fungal but not bacterial diversity and behavioral and cognitive outcomes, whereas both fungal and bacterial loads are associated with worse outcomes. The positive correlations between the bacterial and fungal load measures, and the negative correlations between richness/diversity and the load measures in our study might have contributed to these observations and complicate disentangling effects of the individual microbial measurements.

Our results support the hypothesis that early-life microbial exposure is associated with the development of behavioral problems and cognitive functioning. The hypothesized mechanism for this relationship is through the close connection between cognitive and immune development [16–18]. Consequently, indoor microbiota might influence our behavioral and cognitive functioning through modulation of the immune system in early life [55]. Indeed, exposure to environmental microbiota, in particular exposure to inhalable microbial agents as present in settled dust, is known to be involved in the development of our immune system [13, 14]. Previous research has, for instance, established the link between diversity and specific taxa compositions of the indoor microbial environment and the development of asthma [6–10]. Besides being explicitly involved in regulating our immune system, environmental microbial diversity might play a role in shaping the human microbiome, such as the gut microbiota, which can utilize our immune system and produce neurotransmitters influencing brain regulation [13, 56, 57]. The human gut microbiota are similarly connected to our cognitive development through immunomodulation, resulting in an analogous beneficial influence of microbial diversity on cognitive performance, including improved response times and visuospatial working memory [58–61]. However, it is important to note that without detailed immunological information can only speculate that a well-developed immune system that follows from a highly diverse microbial environment and the environmental contribution to the gut microbiota are driving the associations.

We acknowledge some study limitations. First, cognitive testing in young children can be slightly more difficult to interpret; however, our results were robust to sensitivity analyses accounting for potential age-related problems, including trouble understanding the task or having a potential lack of motivation [43]. Moreover, all cognitive tests were performed in a standardized manner with trained researchers, limiting inter-observer bias and improving reliable data collection. Second, we only used the parent-reported SDQ to assess child behavior. This has, however, proven to be a reliable and validated questionnaire demonstrated to be associated with academic performance in later life [39–41, 62]. In addition, we cannot exclude the possibility of residual confounding. It is possible that certain unaccounted lifestyle characteristics or environmental exposures could modify the indoor microbial exposure, and influence the behavioral and cognitive outcomes. We did, however, perform multiple analyses adjusting for a variety of potential confounders and found only limited changes to the observed associations, supporting the robustness of our findings. Next, we did not investigate the taxa abundances or the microbial profiles that might help explain the underlying processes; however, the observed associations regarding microbial diversity and load are relevant for public health and consistent with studies focusing on immunology. While we acknowledge that the microbial and cognitive assessments were done not at the exact same time, the time difference between those assessments was in all limited to a very large degree and analysis adjusting for this time difference did not change our results (data not shown). While an ideal home microbial exposure assessment would have included multiple sampling points throughout early childhood, we implemented long-term (four to nine weeks) integrated sampling of settling, airborne dust for qualitative and quantitative measurements of indoor microbiota, which avoids issues related to the known short-term variability of indoor air microbial exposures and which is considered to be more representative of indoor microbial exposure over time [63, 64]. Nevertheless, we cannot exclude the possibility that the indoor microbial environment changes over time and that the measured microbial exposure is not fully reflective of early-life indoor microbial exposure. Apart from these restrictions, we provide novel evidence that the indoor microbiota might influence behavioral and cognitive function in young children aged 4–6 years in a large number of households (*n*=172), whilst adjusting for potential confounders and performing sensitivity analyses. We used a comprehensive quantitative and qualitative microbial approach combined with complementary detailed behavioral and cognitive outcomes. Given that our study did not collect sufficient immunological information, we cannot further speculate on the predominant mechanisms. More research is needed to investigate the potential mechanism by which the indoor air-associated microbiota are associated with childhood cognitive function.

## Conclusion

Our findings provide evidence that the early-life microbial environment may play a role in behavior and cognitive function. More specifically, our results suggest that high indoor fungal diversity may be beneficial to behavioral outcomes, and particularly to hyperactive behavior, as well as improved attention and psychomotor speed. In contrast, exposure to high airborne microbial loads could potentially result in worse behavioral and cognitive outcomes. These findings are comparable to the known relationship between early-life exposure to a highly diverse environment and reduced allergies, supporting immunomodulation as a potential mechanism that connects microbiota and cognition. Nevertheless, we need further research to better understand the potential mechanisms involved in the associations observed in this study.

## Supplementary Information

**Additional file 1: Supplemental Figure 1.** Overview of the participation flowchart and exclusion steps. Abbreviations: SDQ, Strengths and Difficulties Questionnaire; CANTAB, Cambridge Neuropsychological Test Automated Battery; BLC, Big/Little Circle; DMS, Delayed matching to sample; MOT, Motor screening Task; SSP, Spatial Span. **Supplemental Figure 2.** Spearman correlation coefficients between CANTAB outcomes of the four tasks (MOT, Motor Screening Task; BLC, Big/Little Circle task; SSP, Spatial Span task and the DMS, Delayed Matching to Sample task). **Supplemental Figure 3.** Spearman correlation coefficients between the bacterial and fungal diversity indices (Chao1 richness, Shannon and Simpson diversity) and loads in house dust samples (Gram-negative bacterial, Gram-positive bacterial and Fungal load). **Supplemental Table 1.** Adjusted* associations (estimate and 95%CI) of microbial diversity indices (Chao1 and Shannon) and loads (Gram-negative bacterial load, Gram-positive bacterial load and Fungal load) with CANTAB variables of the domain of attention and psychomotor speed (Motor Screening Task and Big/Little Circle test) and the domain of visual working memory (Spatial Span test and Delayed Matching to Sample test). **Supplemental Table 2.** Sensitivity analyses excluding children showing possible disinterest: adjusted* associations of microbial diversity indices (Chao1 and Shannon) and loads (Gram-negative bacterial load, Gram-positive bacterial load and Fungal load) with SDQ variables (OR and 95%CI) of the four SDQ scales : peer relationship, emotional, conduct and hyperactivity and the Total Difficulties Score and with CANTAB variables (estimate and 95%CI) of the domain of attention and psychomotor speed (Motor Screening Task and Big/Little Circle task) and the domain of visual working memory (Spatial Span test and Delayed Matching to Sample task.

## Data Availability

The data that support the findings of this study are available from the corresponding author upon reasonable request.
