# Supplementary material for: Association of indoor dust microbiota with cognitive function and behavior in preschool-aged children
Source: Microbiome. 2023 Jan 2;11:1. doi: 10.1186/s40168-022-01406-9 (PMC9806900; doi:10.1186/s40168-022-01406-9)
Supplement: Supplementary file 2 — Additional file 1: Supplemental Figure 1. Overview of the participation flowchart and exclusion steps. Abbreviations: SDQ, Strengths and Difficulties Questionnaire; CANTAB, Cambridge Neuropsychological Test Automated Battery; BLC, Big/Little Circle; DMS, Delayed matching to sample; MOT, Motor screening Task; SSP, Spatial Span. Supplemental Figure 2. Spearman correlation coefficients between CANTAB outcomes of the four tasks (MOT, Motor Screening Task; BLC, Big/Little Circle task; SSP, Spatial Span task and the DMS, Delayed Matching to Sample task). Supplemental Figure 3. Spearman correlation coefficients between the bacterial and fungal diversity indices (Chao1 richness, Shannon and Simpson diversity) and loads in house dust samples (Gram-negative bacterial, Gram-positive bacterial and Fungal load). Supplemental Table 1. Adjusted* associations (estimate and 95%CI) of microbial diversity indices (Chao1 and Shannon) and loads (Gram-negative bacterial load, Gram-positive bacterial load and Fungal load) with CANTAB variables of the domain of attention and psychomotor speed (Motor Screening Task and Big/Little Circle test) and the domain of visual working memory (Spatial Span test and Delayed Matching to Sample test). Supplemental Table 2. Sensitivity analyses excluding children showing possible disinterest: adjusted* associations of microbial diversity indices (Chao1 and Shannon) and loads (Gram-negative bacterial load, Gram-positive bacterial load and Fungal load) with SDQ variables (OR and 95%CI) of the four SDQ scales : peer relationship, emotional, conduct and hyperactivity and the Total Difficulties Score and with CANTAB variables (estimate and 95%CI) of the domain of attention and psychomotor speed (Motor Screening Task and Big/Little Circle task) and the domain of visual working memory (Spatial Span test and Delayed Matching to Sample task. [file 40168_2022_1406_MOESM1_ESM.docx]

**Indoor dust microbial communities may influence cognitive development in children aged 4 to 6 years**

Yinthe Dockx^a^, Martin Täubel^b^, Janneke Hogervorst^a^, Leen Luyten^a^, Martien Peusens^a^, Leen Rasking^a^, Hanne Sleurs^a^, Katrien Witters^a^, Michelle Plusquin^a^, Maria Valkonen^b^, Tim S Nawrot^a,c^, Lidia Casas^c,d,e^

^a^ Centre for Environmental Sciences, Hasselt University, Agoralaan Building D, 3590 Diepenbeek, Belgium
[yinthe.dockx@uhasselt.be](mailto:yinthe.dockx@uhasselt.be); [janneke.hogervorst@uhasselt.be](mailto:janneke.hogervorst@uhasselt.be); [leen.luyten@uhasselt.be](mailto:leen.luyten@uhasselt.be) ; [martien.peusens@uhasselt.be](mailto:martien.peusens@uhasselt.be) ; [leen.rasking@uhasselt.be](mailto:leen.rasking@uhasselt.be) ; [hanne.sleurs@uhasselt.be](mailto:hanne.sleurs@uhasselt.be) ; [katrien.witters@uhasselt.be](mailto:katrien.witters@uhasselt.be) ; [michelle.plusquin@uhasselt.be](mailto:michelle.plusquin@uhasselt.be) ; [tim.nawrot@uhasselt.be](mailto:tim.nawrot@uhasselt.be)

^b^ Environmental Health Unit, Department Health Security, Finnish Institute for Health and Welfare, Kuopio, Finland

[martin.taubel@thl.fi](mailto:martin.taubel@thl.fi); [maria.valkonen@thl.fi](mailto:maria.valkonen@thl.fi)

^c^ Center for Environment and Health, Department of Public Health, Leuven University (KU Leuven), Herestraat 49 – 706, BE-3000 Leuven, Belgium

[tim.nawrot@uhasselt.be](mailto:tim.nawrot@uhasselt.be) ; [lidia.CasasRuiz@uantwerpen.be](mailto:lidia.CasasRuiz@uantwerpen.be)

^d^ Social Epidemiology and Health Policy, Department of Family Medicine and Population Health, University of Antwerp, Doornstraat 331, 2610 Wilrijk, Belgium

[lidia.CasasRuiz@uantwerpen.be](mailto:lidia.CasasRuiz@uantwerpen.be)

^e^ Institute for Environment and Sustainable Development (IMDO), University of Antwerp, Groenenborgerlaan 171, 2020 Antwerp, Belgium

[lidia.CasasRuiz@uantwerpen.be](mailto:lidia.CasasRuiz@uantwerpen.be)

Correspondence to Yinthe Dockx, Centre for Environmental Sciences, Hasselt University, Agoralaan Building D, 3590 Diepenbeek, Belgium. Telephone: 32-11-268584. E-mail: yinthe.dockx@uhasselt.be

**Supplemental Figures**


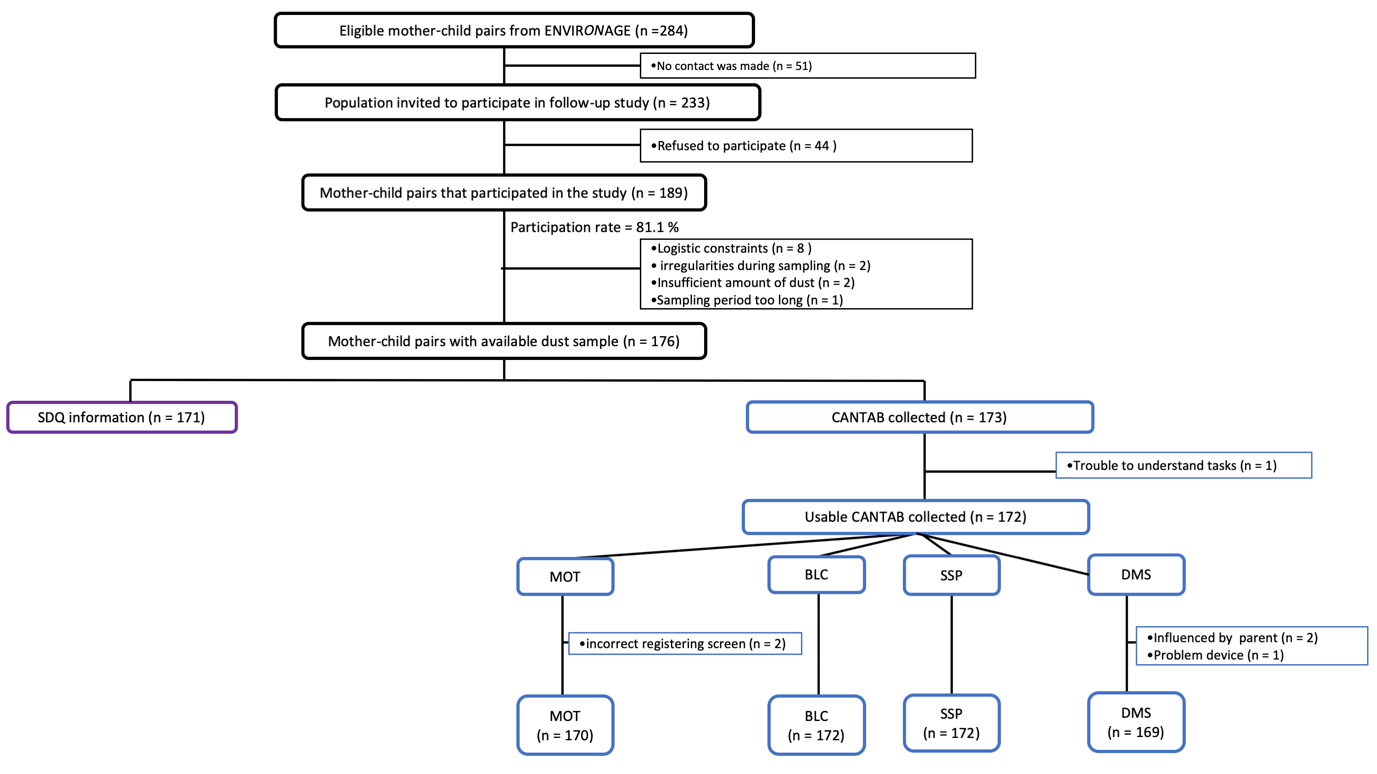


**Supplemental Figure 1** Overview of the participation flowchart and exclusion steps. Abbreviations: SDQ, Strengths and Difficulties Questionnaire; CANTAB, Cambridge Neuropsychological Test Automated Battery; BLC, Big/Little Circle; DMS, Delayed matching to sample; MOT, Motor screening Task; SSP, Spatial Span

**Supplemental Figure 2** Spearman correlation coefficients between CANTAB outcomes of the four tasks (MOT, Motor Screening Task; BLC, Big/Little Circle task; SSP, Spatial Span task and the DMS, Delayed Matching to Sample task).

**Supplemental Figure 3** Spearman correlation coefficients between the bacterial and fungal diversity indices (Chao1 richness, Shannon and Simpson diversity) and loads in house dust samples (Gram-negative bacterial, Gram-positive bacterial and Fungal load).

**Supplemental Table 1 Adjusted* associations (estimate and 95%CI) of microbial diversity indices (Chao1 and Shannon) and loads (Gram-negative bacterial load, Gram-positive bacterial load and Fungal load) with CANTAB variables of the domain of attention and psychomotor speed (Motor Screening Task and Big/Little Circle test) and the domain of visual working memory (Spatial Span test and Delayed Matching to Sample test).**

|  |  | **Microbial Diversity***** | | | | **Microbial load****** | | |
| --- | --- | --- | --- | --- | --- | --- | --- | --- |
|  |  | **Bacteria** | | **Fungi** | | **Gram-negative**  **load** | **Gram-positive load** | **Fungal**  **load** |
|  | | **Chao1** | **Shannon** | **Chao1** | **Shannon** |  |  |  |
| **Attention and psychomotor speed** | |  |  |  |  |  |  |  |
| **Motor Screening Task** | |  |  |  |  |  |  |  |
| Response time** | | 1.35[-3.73;6.71] | 0.37[-4.29;5.26] | -0.42[-5.40;4.82] | -0.86[-6.04;4.61] | -0.88[-3.65;1.97] | -0.51[-3.12;2.18] | 0.99[-1.67;3.73] |
| Error | | 0.56[-0.03;1.14] | 0.08[-0.46;0.63] | -0.13[-0.72;0.46] | -0.06[-0.68;0.56] | 0.06[-0.27;0.39] | 0.05[-0.26;0.35] | -0.00[-0.31;0.31] |
| **Big/Little Circle** | |  |  |  |  |  |  |  |
| Response time** | | -0.82[-3.64;2.08] | -0.02[-2.65;2.69] | -1.78[-4.53;1.05] | **-3.20[-6.01;-0.30]** | 0.12[-1.43;1.69] | 0.47[-0.97;1.94] | 1.20[-0.27;2.68] |
| **Visual working memory** | |  |  |  |  |  |  |  |
| **Spatial Span** | |  |  |  |  |  |  |  |
| Span length | | 0.17[-0.07;0.42] | 0.19[-0.03;0.42] | 0.14[-0.11;0.38] | -0.10[-0.36;0.16] | -0.05[-0.18;0.09] | -0.02[-0.14;0.11] | -0.01[-0.14;0.12] |
| **Delayed Matching to Sample** | |  |  |  |  |  |  |  |
| Response time** | | 1.86[-6.27;10.68] | 6.34[-1.38;14.67] | -4.81[-11.99;2.97] | -1.44[-9.78;7.66] | **5.74[1.04;10.67]** | **5.17[0.87;9.65]** | **5.70[1.44;10.14]** |
| Probability of error | | -0.06[-4.67;4.56] | -0.88[-5.01;3.25] | 0.71[-3.68;5.09] | -2.94[-7.52;1.64] | -0.31[-2.70;2.08] | -0.03[-2.26;2.21] | 0.70[-1.60;2.99] |
| Percentage correct | | -0.34[-3.80;3.13] | 0.36[-2.78;3.50] | -0.72[-4.02;2.57] | 1.66[-1.80;5.11] | 0.65[-1.15;2.45] | 0.91[-0.79;2.60] | -0.25[-1.98;1.48] |

* Associations adjusted for child’s sex, age, maternal education, time of examination, urbanicity and sampling duration. Estimates presented in bold are statistically significant p < 0.05.

**Response times are expressed as a percentage change.

*** Associations with microbial diversity indices are expressed for IQR increments

***Associations with microbial load are expressed for a 2-fold increment

**Supplemental Table 2** **Sensitivity analyses excluding children showing possible disinterest:** **adjusted* associations of microbial diversity indices (Chao1 and Shannon) and loads (Gram-negative bacterial load, Gram-positive bacterial load and Fungal load) with SDQ variables (OR and 95%CI) of the four SDQ scales : peer relationship, emotional, conduct and hyperactivity and the Total Difficulties Score and with CANTAB variables (estimate and 95%CI) of the domain of attention and psychomotor speed (Motor Screening Task and Big/Little Circle task) and the domain of visual working memory (Spatial Span test and Delayed Matching to Sample task.**

|  |  | **Microbial Diversity**** | | | | **Microbial load***** | | |
| --- | --- | --- | --- | --- | --- | --- | --- | --- |
|  |  | **Bacteria** | | **Fungi** | | **Gram-negative**  **load** | **Gram-positive load** | **Fungal**  **load** |
|  | | **Chao1** | **Shannon** | **Chao1** | **Shannon** |  |  |  |
| **Attention and psychomotor speed** | |  |  |  |  |  |  |  |
| **Motor Screening Task** | |  |  |  |  |  |  |  |
| Response time | | 1.40[-3.70;6.78] | 0.37[-4.32;5.30] | -0.30[-5.29;4.96] | -0.87[-6.06;4.60] | -0.79[-3.55;2.06] | -0.53[-3.14;2.15] | 0.85[-1.81;3.59] |
| Error | | 0.56[-0.03;1.15] | 0.08[-0.47;0.64] | -0.13[-0.73;0.47] | -0.06[-0.69;0.56] | 0.06[-0.27;0.39] | 0.05[-0.26;0.35] | -0.00[-0.31;0.31] |
| **Big/Little Circle** | |  |  |  |  |  |  |  |
| Response time | | -0.76[-3.62;2.19] | -0.06[-2.72;2.68] | -1.71[-4.47;1.12] | **-3.21[-6.02;-0.32]** | 0.17[-1.39;1.75] | 0.43[-1.03;1.90] | 1.10[-0.37;2.60] |
| **Visual working memory** | |  |  |  |  |  |  |  |
| **Spatial Span** | |  |  |  |  |  |  |  |
| Span length | | 0.19[-0.08;0.45] | 0.18[-0.06;0.42] | 0.17[-0.08;0.41] | -0.07[-0.33;0.20] | -0.06[-0.20;0.08] | -0.03[-0.15;0.10] | -0.03[-0.16;0.10] |
| **Delayed Matching to Sample** | |  |  |  |  |  |  |  |
| Response time | | 0.86[-7.16;9.58] | 5.54[-2.33;14.04] | -5.60[-12.97;2.40] | -2.24[-10.50;6.79] | **6.29[1.57;11.24]** | **5.56[1.25;10.06]** | **5.98[1.73;10.40]** |
| Probability of error | | -0.88[-5.51;3.74] | -2.32[-6.50;1.87] | 0.51[-3.86;4.87] | -3.24[-7.78;1.29] | 0.27[-2.13;2.66] | 0.52[-1.71;2.76] | 0.94[-1.32;3.194] |
| Percentage correct | | 0.29[-3.20;3.77] | 1.43[-1.76;4.62] | -0.42[-3.71;2.86] | 1.98[-1.45;5.40] | 0.17[-1.64;1.98] | 0.48[-1.22;2.18] | -0.44[-2.14;1.26] |

* Associations adjusted for child’s age, sex, maternal education, time of examination*,* urbanicity, sampling duration. Estimates presented in bold are statistically significant p < 0.05.

** Associations with microbial diversity indices are expressed for IQR increments

***Associations with microbial load are expressed for a 2-fold increment

**The PCR procedure, sequencing, sequence processing and bioinformatics analyses**

ITS and 16S PCR, fungal and bacterial 16S amplicon sequencing

The DNA extracted from dust and control samples was shipped frozen to the sequencing service partner LGC Genomics (Germany), who did the library preparation and sequencing. The V4 region of the bacterial 16S rRNA gene was amplified using 515F/806R primers^[^[^34^](#_ENREF_34)^]^. For fungi, the ITS1 region of the Internal Transcribed Spacer (ITS) was amplified using ITS1F/ITS2 primers.^[^[^35^](#_ENREF_35)^]^

The PCRs included about 1-10 ng of DNA extract (total volume 1μl), 15 pmol of each forward and reverse primer in 20 μl volume of MyTaq buffer containing 1.5 units MyTaq DNA polymerase (Bioline GmbH, Luckenwalde, Germany) and 2 μl of BioStabII PCR Enhancer (Sigma-Aldrich Co.). For each sample, the forward and reverse primers had the same 10-nt barcode sequence. PCRs were carried out for 35 cycles using the following parameters: 1 min 96 °C predenaturation; 96 °C denaturation for 15 s, 55 °C annealing for 30 s, 70 °C extension for 90 s, hold at 8 °C. DNA concentration of amplicons of interest was assessed by gel electrophoresis. About 20 ng amplicon DNA of each sample were pooled for up to 48 samples carrying different barcodes. The amplicon pools were purified with one volume Agencourt AMPure XP beads (Beckman Coulter, Inc., IN, USA) to remove primer dimer and other small mispriming products, followed by an additional purification on MinElute® columns (QIAGEN GmbH, Hilden, Germany). About 100 ng of each purified amplicon pool DNA was used to construct Illumina libraries using the Ovation® Rapid DR Multiplex System 1–96 (NuGEN Technologies, Inc., CA, USA). Illumina libraries (Illumina, Inc., CA, USA) were pooled and size selected by preparative gel-electrophoresis.

Sequencing was performed on an Illumina MiSeq with V3 chemistry resulting in paired-end reads with a length of 300 bp each. The libraries were demultiplexed using Illumina’s bcl2fastq v1.8.4 (https://support.illumina.com/downloads/bcl2fastq_conversion_software_184.html) and all sequence reads processed with custom Python v2.7.6 scripts to sort them by sample, removing barcode and amplicon primer sequences. Adapter sequences were removed from the 3′ end of reads with a proprietary script discarding reads shorter than 100 bp.

Sequence processing and bioinformatics analyses

16S and ITS amplicon data was analyzed by standard dada2 pipeline version 1.8.^[^[^36^](#_ENREF_36)^]^ The pipeline inputs the forward R1 and reverse reads R2 in fastq format files and process the data with removal of bad quality reads based on the read quality profile. Then, the filtering and trimming step was carried out applying default parameters in the filterAndTrim function with truncLen parameter for bacteria (200,200) and without truncLen parameter for fungi due to variation in ITS region. De-replication of reads was done followed by inference of ASV using dada2 core sample inference algorithm. Further, merging of forward and reverse reads were merged together to form contigs with overlap of 12 nucleotides. Then, the ASV table with sequence count was constructed with rows as ASV and columns as samples. Sequences that were longer or shorter than the expected length due to non-specific priming were removed. Finally, chimeras were removed and the final ASV table was constructed for further downstream processing. Taxonomy was assigned using SILVA^[^[^37^](#_ENREF_37)^]^ database version 132 for bacteria and UNITE database version 7.2 for fungi^[^[^38^](#_ENREF_38)^]^. For bacterial data, phylogenetic tree was constructed in QIIME version 1.9.1^[^[^39^](#_ENREF_39)^]^ with the ASV sequences for the tree based diversity calculations. The downstream post processing included removal of singletons, mitochondria, eukaryote and chloroplast ASVs. In order to flag and remove potential contaminants from the sequence datasets, we included sequencing of 14 negative and reagent controls from DNA extraction (alongside bacterial and fungal mock communities), as well as extracts of four petri dish blank samples. In order to flag and remove contamintant ASVs from the bacterial data we applied function isContaminant of the Decontam package version 1.2^[^[^69^](#_ENREF_69)^]^, using “prevalence” method and probability threshold 0.1. This method compares prevalence of detection of an ASV in controls versus real samples and flags ASVs that occur at higher prevalence in the controls, as to be expected from reagent and other contaminants. Using this approach, a total of 81 bacterial ASVs were removed prior to downstream analyses (Supplemental Table 6). In the fungal data, ASVs detected in samples occurred only sporadically, no more than once, in any of the 14 negative controls, and none of those four ASVs (Supplemental Table 7) were identified in any of the four blanks. For these reasons, these ASVs were not considered true contaminants and were kept in the dataset. Samples with less than 1000 sequences (n=13 samples for bacteria and 19 samples for fungi, respectively) as well as mock communities were excluded from downstream analyses. The alpha diversity within samples – including Chao1, observed ASVs, Shannon, Simpson metrics were calculated in QIIME, applying rarefaction values of 1495 sequences for bacteria and 3956 sequences for fungi, respectively. Taxa summary at different taxonomic levels were constructed using summarize_taxa_through_plots.py script in QIIME pipeline.

**Cognitive assessment**

To assess the child’s cognitive function, we used the CANTAB^[28]^ software on a touch screen tablet, which is deemed reliable for measuring executive functions in children as young as four years of age.^[36]^ Before cognitive testing, the child was told that each test could be stopped at all times if they were feeling uncomfortable or scared. Subsequently, a trained examiner gave standardized instructions at the beginning of each task according to a script provided by the software developers. This was repeated once if the child did not seem to understand the task or was unsure. Each test had test trials which were not used to calculate the outcome variables. In total, the child was given four tasks to complete, of which the first two were designed to assess the attention and psychomotor speed (the Motor Screening task and the Big/Little Circle task) and the last two were used to measure the child’s visuospatial working memory (Spatial Span test) and visual short-term recognition memory (Delayed Matching to Sample task).

Attention and psychomotor speed

The first test assessing the attention and psychomotor speed was the Motor Screening Task (estimated administration time: 2 minutes) , in which the child had to press the screen as quickly and accurately as possible on a series of ten crosses at differing positions on the screen. The cross was deemed successfully pressed if the touch was within a close radius of the cross’s center (based on a screen resolution of 640 x 350 pixels).

The next cognitive test for assessing the attention and psychomotor speed was the Big/Little Circle task (estimated administration time: 2 minutes). The child was first asked to select the smallest circle of two differently sized circles as quickly as possible in a series of 20 circle pairs. Afterwards, the child was presented with another series of 20 circle pairs, of which the child was asked to select the largest circle.

visual working and recognition memory

In the Spatial Span test (estimated administration time: 5 minutes), the child was presented with ten white squares that would briefly change color in a randomized sequence. The child had to reproduce the sequence after an auditory cue and if repeated correctly the sequence was increased by one, starting from a sequence of two colored squares. The test was completed if the child had failed to reproduce the sequence after three consecutive errors within the same sequence length. In the fourth and last test, i.e., Delayed Matching to Sample task (estimated administration time: 7 minutes), the child was first presented with a complex visual pattern that would be used as the sample pattern to recognize and recall from four similar patterns that were randomly shown either simultaneously or after a brief delay (0, 4, 12 seconds) after which the sample pattern disappeared, repeated for 20 trials. The patterns the child had to choose from consisted of one being identical to the sample pattern, one being identical except for the shapes, one being identical except for the colors, and one having both different shapes and colors.
